# Morphological and geochemical variations of *Cyprideis* (Ostracoda) from modern waters of the northern Neotropics

**DOI:** 10.1007/s10201-016-0504-9

**Published:** 2016-10-06

**Authors:** J. Meyer, C. Wrozyna, M. Gross, A. Leis, W. E. Piller

**Affiliations:** 1 Institute of Earth Sciences, University of Graz, NAWI Graz Geocenter, Heinrichstrasse 26, 8010 Graz, Austria; 2 Geology and Palaeontology, Universalmuseum Joanneum, Weinzöttlstrasse 16, 8045 Graz, Austria; 3 Joanneum Research; Resources, Institute of Water, Energy and Sustainability, Elisabethstrasse 18/II, 8010 Graz, Austria

**Keywords:** Ostracods, Stable isotopes, Neotropics, *Cyprideis*, Morphology

## Abstract

The variability of modern *Cyprideis salebrosa* and *Cyprideis americana* (Ostracoda) from the northern Neotropics were investigated in order to understand site specific influences on the isotopic composition of their valves (δ^18^O, δ^13^C) in comparison to their host water and to connect this to morphological features of their valves (valve size, nodosity). *C. salebrosa* was found in a stream (Shell Creek, Florida) and a slightly brackish lake (Laguna del Rincon, Dominican Republic; salinity <0.7 psu) while *C. americana* occurred in a coastal lake with polyhaline waters (Parrotee Pond, Jamaica; salinity: >20 psu). Valve size and position of nodes differed between the two species. A reverse temperature dependency have been considered to influence Shell length (seasonally in Shell Creek, summer: 1076 µm; winter: 1092 µm, supposedly permanently in Laguna del Rincon, 1035 µm). But, regarding the small dataset other factors could not be excluded to influence ostracod valve size. A decline of node frequency of *C. salebrosa* is mainly related to an increase in salinity. Isotopic values of ostracod valves reflect the trend in stable isotopes of their host water. Variations in *Cyprideis salebrosa* δ^18^O and δ^13^C values signify differences in their host water. Offsets of ostracod valves to a theoretical calcite precipitated in their host water with an uncertain time lag (+0.015 to +2.63 ‰) needs to be clarified. This study presents a contribution to the understanding of environmental influences on modern ostracod shell characters as basis for paleontological applications.

## Introduction

Morphological characteristics as well as stable isotopic signatures of ostracod valves are commonly used to reconstruct environmental conditions (e.g., Frenzel and Boomer 2005). Such proxies provide information on different environmental parameters (e.g., salinity, temperature, hydrochemistry) and the combined investigation of these characteristics can be important to understand specific conditions (e.g., hydrology) of the habitat. Investigations of morphological characteristics of ostracod valves are based on the observations of single valves while for stable isotopes often multiple shell measurements are possible only. But, intra-sample variability of multiple single valve measurements can track high-frequency variations that give information on short term variability of an environment (Escobar et al. 2010).

In this context, investigations on modern ostracods and synchronously of their host water are crucial to calibrate these proxies.

*Cyprideis* is a suitable genus investigating morphological and isotopic characteristics of single valves related to environmental conditions. It is known to live in freshwater to hyperhaline environments and different *Cyprideis* species have a diverging salinity range (Sandberg 1964; Keyser 1978). Within its environmental constraints, morphological characteristics of a single species can vary notably. Extant members of the genus *Cyprideis* in the Americas were studied by Sandberg (1964) including detailed descriptions of morphological characteristics and their phenotypic plasticity. For instance, noding on the valves of *Cyprideis torosa* (Jones) (and also other species of *Cyprideis*) increases when salinity decreases (Sandberg 1964; Van Harten 1975, 2000). The size, shape and sieve pores of *Cyprideis* valves have also been reported to vary with different environmental parameters (e.g., Van Harten 1975; Schweitzer and Lohmann 1990; Bowles 2013). Data on isotopic signatures of recent valves from that genus only exists for *C. torosa* from the Iberian Peninsula with site specific isotopic compositions (Marco-Barba et al. 2012).

Other studies on the isotopic composition of ostracod valves demonstrated, that they are not in isotopic equilibrium with the surrounding water. In general, valves have a higher ^18^O content compared to an inorganic calcite grown in equilibrium under the same conditions (Xia et al. 1997a; von Grafenstein et al. 1999; Keatings et al. 2002; Li and Liu 2010; Decrouy et al. 2011). Most of these studies are based on monthly sampling or defined laboratory conditions. Such monitoring allows the connection of isotopic data from ostracod valves with water and temperature data from their calcification period. But, direct monitoring is not always possible and time offsets cannot be corrected. Nevertheless, it has been shown that the approach of simultaneous measurments of recent ostracods and their host water provide reliable and significant results (Wetterich et al. 2008; Van der Meeren et al. 2011).

Studies on the isotopic composition of the modern freshwater ostracods are, however, still rare in the Neotropics. One of the few surveys using isotopes of modern ostracods to interpret the stable isotope record of ostracods from a sediment core was presented by Pérez et al. (2013) for Lago Petén Itzá (Guatemala).

The objectives of this study were (1) to explore environmentally induced differences in morphology and the isotopic composition of modern ostracod valves on a spatial scale, and (2) to investigate how water conditions influence within sample variability of isotopic signatures on a short time scale. Herein, we present data of two *Cyprideis* species as well as geochemical and isotopic data of their host waters from modern waters in Florida, the Dominican Republic and Jamaica.

## Study area

**Fig. 1:**
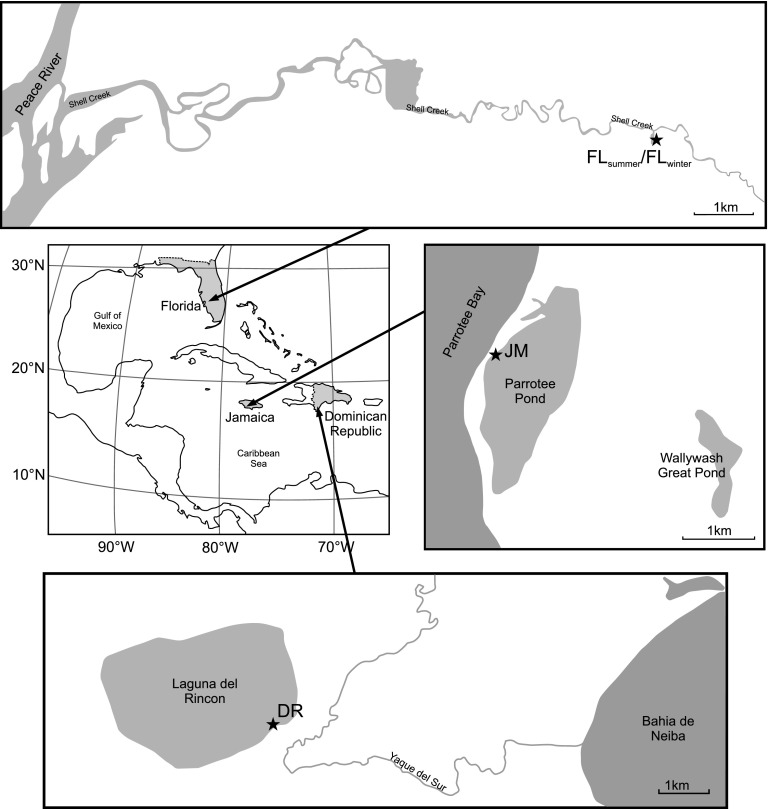
Location of the three studied sites from Shell Creek in Florida (FL_summer_/FL_winter_), Laguna del Rincon in the Dominican Republic (DR) and Parrotee Pond in Jamaica (JM). Sample locations are marked by *black stars*

Study sites include a location at Shell Creek in Florida (USA) (26°58′27.04″N 81°53′21.55″W), Laguna del Rincon in the Dominican Republic (18°16′7.03″N 71°13′9.74″W) and Parrotee Pond in Jamaica (17°59′2.69″N 77°50′14.70″W) (Fig. 1).

A water sample and a surface sediment sample were taken from the littoral zone of an artificial dead end branch of Shell Creek (FL_winter_), from the littoral zone of Laguna del Rincon (DR) and in a shallow water area (~20–30 cm) of Parrotee Pond (JM) in November/December 2013, respectively. Sampling in the littoral zone of Shell Creek was repeated in August 2014 at the same site (FL_summer_).

Shell Creek is a small river in the southwest of Florida. It is part of the Peace River catchment and originates in the northwest of Charlotte County. It flows westward to the Gulf of Mexico where it converges with the Peace River at Harbour Heights. A station from the U.S. Geological Survey (02297635 Shell Creek on CR 764 near Punta Gorda, FL) is located 200 m from the investigated site providing daily information on water temperature and electrical conductivity (EC) for 2013 (U.S. Geological Survey 2014) and 2014 (online at the USGS National Water Information System (NWIS)). Water temperature varied within that period between 15.1 and 30.8 °C with highest temperatures in June to August and lowest in January. The EC varied between 125 and 1460 µS/cm with highest values between January and April and lowest between June and September.

In general, Florida is characterized by a subtropical climate. Mean air temperature ranges from 20 °C in January to 27.5 °C in August from 1971 to 2000 (Price et al. 2008). It has a wet season from May to September with maximum precipitation in August (205.7 mm) and minimum precipitation in October (9.4 mm) (Kane and Dickman 2006).

Laguna del Rincon is located in the southwest of the Dominican Republic. The lake lies southeast of Lago Enriquillo, about 11.5 km off the coast. The Lago Enriquillo Valley was isolated from the Caribbean Sea by tectonic uplift and fluvial damming about 5000–2800 years ago (Mann et al. 1984). Since this time the valley is a closed basin whose hydrology is primarily shaped by evaporation (Buck et al. 2005). Today the only inflows to Laguna del Rincon are small artificial, rainwater fed channels around the lake. Data near Santo Domingo from the Global Network for Isotopes in Precipitation (GNIP) show temperatures between 25.0 °C in February and 28.5 °C in August between 1996 and 2002 and a wet season between May and October with maximum precipitation in August or September (<350 mm) (http://isohis.iaea.org). There is a dry season from December to April with average rainfalls lower than 20 mm (Buck et al. 2005).

Parrotee Pond is a shallow brackish water lagoon in the southwest of Jamaica, about 50 m off the shoreline. A coastal brackish aquifer underlies the pond and, presumably, influences it by mixing with marine groundwater (UNDP/FAO 1971). The pond has no overland inflow and is mainly fed by annual precipitation. Precipitation at Black River (northward of Parrotee Pond) ranges from 43 mm in January to 207 mm in October (Nkemdirim 1979). Mean monthly air temperature of Jamaica reaches 25.8 °C in January and 29.0 °C in July (Curtis et al. 2014).

## Materials and methods

### Sediment samples and water analyses

Surface sediment samples were taken using a hand-net, sieved at the laboratory (fractions: ≥2, ≥1 mm, ≥500, ≥250, ≥125, ≥63 µm) and stored in ethanol (96 %) immediately.

Simultaneously to water sampling, field variables (electrical conductivity, water temperature and pH) were measured in situ at all sample sites using a multi-sensor probe. Water samples were promptly filtrated using a syringe filter with a filter pore size of 0.45 µm and stored until analysis.

Major ions, the isotopic composition of the water (δ^18^O, δD) and dissolved inorganic carbon (δ^13^C_DIC_) were measured at the laboratory center of Joanneum Research in Graz. Major ions were determined by ion chromatography (Dionex ICS-3000). The hydrogen and oxygen isotope ratios of freshwater were analysed by wavelength-scanned cavity ring-down spectroscopy (WS-CRDS) using a L2120-I system from Picarro coupled with an evaporator and a liquid autosampler. The analytical procedure that was used in this study is similar to the method described by Brand et al. (2009). The oxygen isotopic composition of brackish water was measured by using the classic CO_2_–H_2_O equilibrium technique (Epstein and Mayeda 1953) with a fully automated device adapted from Horita et al. (1989) coupled to a Finnigan DELTA^plus^ Dual Inlet Mass Spectrometer. The stable isotopes of hydrogen in brackish water were measured using a Finnigan DELTA^plus^ XP mass spectrometer working in continuous flow mode by the chromium reduction method (Morrison et al. 2001).

For stable carbon isotope analyses (^13^C_DIC_) approximately 1 ml solution was injected into a 10 ml gas tight vial. Previously to sampling the vials were flushed with helium gas and preloaded with six droplets of phosphoric acid in the Lab. Isotopic composition of DIC was analysed using a Gasbench II device (Thermo) which was connected to a Finnigan DELTA^plus^ XP isotope ratio mass spectrometer. Analytical setup is comparable to that used in other studies (Spötl 2005). Results of water and DIC measurements are given in per mil (‰) with respect to Vienna Mean Ocean Water (V-SMOW) and Vienna Peedee Belemnite (V-PDB), respectively, using the standard delta notation. The analytical precision for stable isotope measurements is ± 0.8 ‰ for δD, ± 0.08 ‰ for δ^18^O in water and ± 0.1 ‰ δ^13^C in DIC.

### Ostracod material

Ostracods were picked from the sediment samples under a binocular (Zeiss Discovery V8) and valves of *Cyprideis salebrosa* Van den Bold, 1963 and *Cyprideis americana* (Sharpe, 1908) were separated and stored in micro slides for isotopic measurements. *C. salebrosa* was identified in accordance with the first description of the species by Van den Bold (1963) and further detailed descriptions provided by Sandberg (1964). *Cyprideis americana* was originally described as *Cythere americana* by Sharpe (1908) but later transferred to the genus *Cyprideis* by Sandberg (1964; for additional remarks see Sandberg and Plusquellec 1974). Our specimens from Parrotee Pond fit well with the material of Sharpe (1908), Sandberg and Plusquellec (1974) and Bowles (2013).

Morphometric analyses on the valves included length and height measurements and record of nodes (number and position).

#### Isotope analyses

Different numbers of ostracod valves per sample were measured for carbon and oxygen stable isotopes at the Institute of Earth Sciences, University of Graz. The number of individual measurements ranged from 42 to 79 valves per sample depending on the availability in the samples. The samples were reacted with 100 % phosphoric acid at 70 °C in a Kiel II automated reaction system and measured with a Finnigan DELTA^plus^ isotope-ratio mass spectrometer. Reproducibility of replicate analyses for standards (in-house and NBS 19) was better than ±0.08 ‰ for δ^13^C and ±0.1 ‰ for δ^18^O. All carbonate isotopic values are quoted relative to V-PDB.

Valves of adult individuals and individuals of the last juvenile stage (A-1) were used for isotopic measurements. Adult individuals (male and female) provided enough material to perform stable isotope analysis on single valves. For the juvenile specimens, two valves per measurement were used. Valves of eight individuals of *C. salebrosa* from the Dominican Republic were tested for the similarity of isotopic values in right and left valves. Despite no difference was detected, only left valves were used for isotopic measurements.

Prior to isotopic analyses possible contaminations were removed from all ostracod valves with deionised water, brushes and entomological needles. To remove stronger contaminations single valves were kept in 10 % H_2_O_2_ for 5–10 min at room temperature and cleaned with deionised water and brushes afterwards. Furthermore, to exclude a shift in the isotopic values of the valves due to H_2_O_2_ treatment, right valves of *C. salebrosa* from FL_winter_ were stored in 10 % H_2_O_2_ at room temperature for 5, 10, 30, 120 and 240 min, respectively, and stable isotopes were measured afterwards. Left valves of the same individuals stayed untreated and were also measured separately.

According to different degrees of preservation, the shell material was classified into three categories: fresh (valves were open with well-defined soft parts in it), neutral (valves without soft parts and no signs of dissolution) and altered (valves with signs of dissolution and discolorations). Valves of all three categories were measured for isotopes.

#### Calculation of isotope fractionation factors

To describe the fractionation of oxygen isotopes measured from ostracod valves in comparison to calcite grown under equilibrium the isotopic fractionation factor of two chemical phases (*α*_*X*–*Y*_) was calculated. Phases *X* and *Y* represent the measured isotopic values of the ostracod calcite (*X*) and their host water (*Y*).

1 $$\propto_{\text{calcite - water}} = (1000 + \delta^{18} {\text{O}}_{\text{calcite}} )/(1000 + \delta^{18} {\text{O}}_{\text{water}} )$$

The δ^18^O values of both calcite and water in the equation above are expressed relative to V-SMOW, whereas our own values for calcite and water are expressed relative to V-PDB and V-SMOW, respectively. To convert the δ^18^O values, the expression of Coplen et al. (1983) was used:

2 $$\delta^{18} {\text{O}}_{\text{V - SMOW}} = 1.03091 \times \delta^{18} {\text{O}}_{\text{V - PDB}} + 30.91.$$

## Results

### Water chemistry and stable isotope composition

**Fig. 2:**
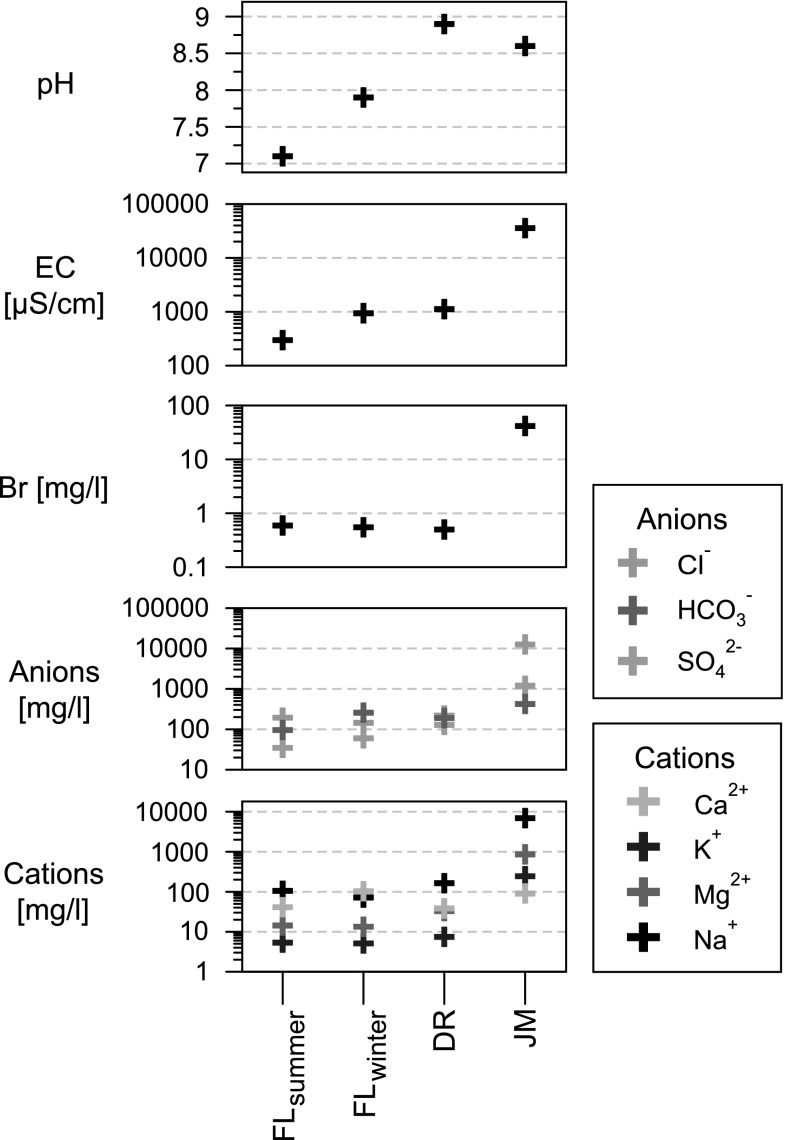
Physico-chemical values of the water samples from the three studied sites. *FL*_*summer*_ summer sample Shell Creek, Florida; *FL*_*winter*_ winter sample Shell Creek, Florida; *DR* Laguna del Rincon, Dominican Republic; *JM* Parrotee Pond, Jamaica; *EC* electrical conductivity; *Br* bromide, as indicator for saltwater mixing

**Fig. 3:**
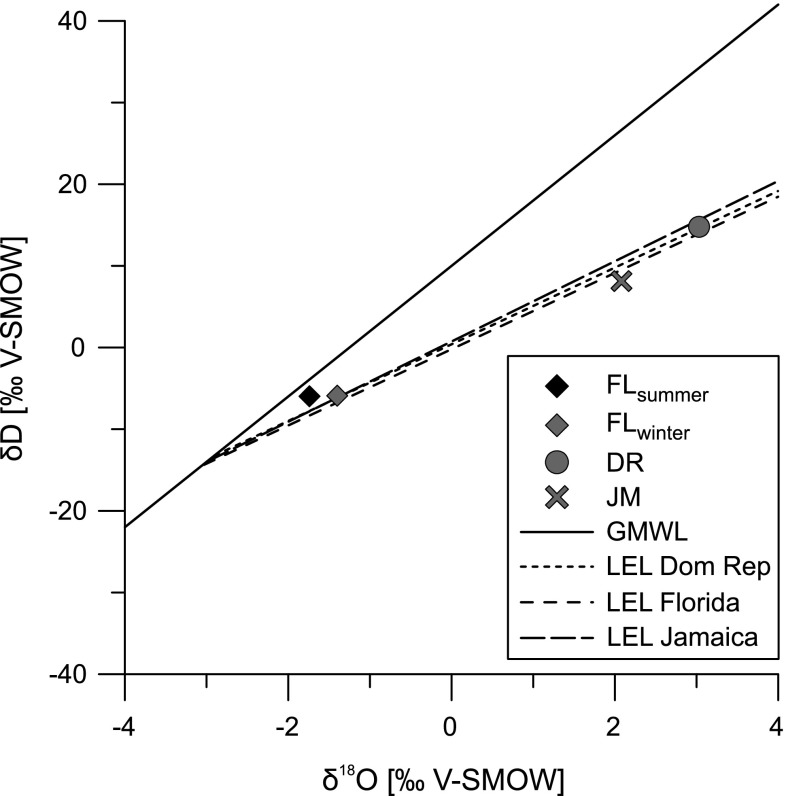
Stable oxygen and deuterium isotope composition of all water samples in comparison to the Global Meteoric Water Line (GMWL) (Craig 1961) and Local Evaporation Lines (LEL) from Florida (Sacks 2002), Dominican Republic (Buck et al. 2005) and Jamaica (Holmes et al. 1995)

**Fig. 4:**
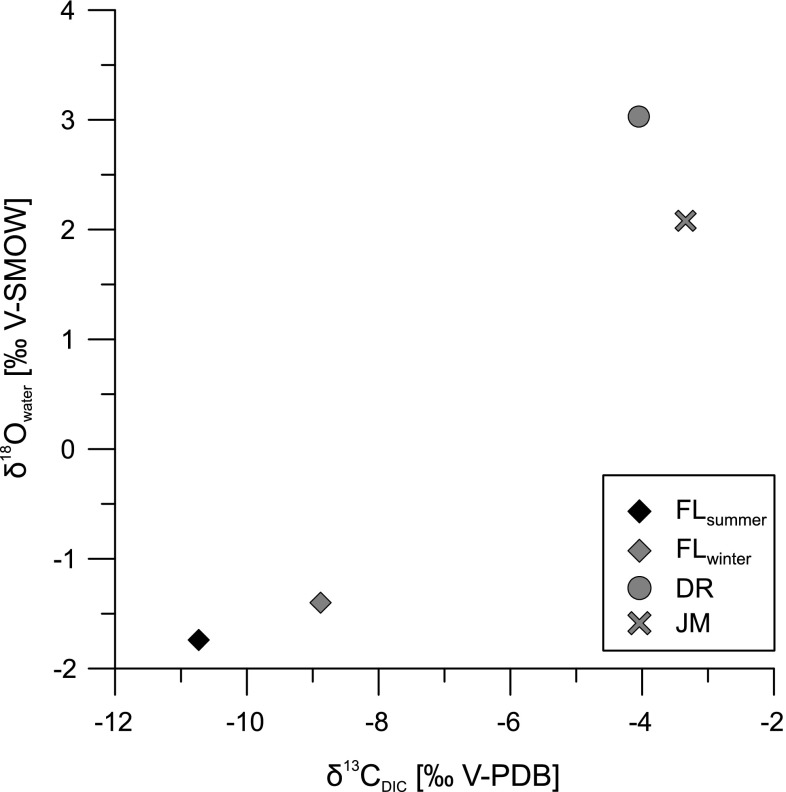
Stable carbon isotopes of dissolved inorganic carbon and stable oxygen isotopes of the investigated water samples

All measured and analyzed parameters from all study sites are presented in Table 1 and Figs. 2, 3, 4.

**Table 1:** Field parameters, isotopic and ionic composition of collected water samples from Jamaica, the Dominican Republic and Florida in 2013 and 2014

Sample			Field parameters			Stable isotopes			Cations				Anions				SI_calcite_^a^
Name	Date	Location	Cond. [µS/cm]	Temp. [°C]	pH	δD [‰ V-SMOW]	δ^18^O [‰ V-SMOW]	δ^13^C_DIC_ [‰ V-DPB]	Na [mg/l]	K [mg/l]	Mg [mg/l]	Ca [mg/l]	Cl [mg/l]	Br [mg/l]	SO_4_ [mg/l]	HCO_3_ [mg/l]	
FL_summer_	08.08.14	Shell Creek, Florida	297	31.2	7.1	−6.0	−1.74	−10.73	105.25	5.35	14.31	40.99	193.77	0.59	34.74	95.80	−0.70
FL_winter_	28.11.13	Shell Creek, Florida	938	20.3	7.9	−5.9	−1.40	−8.88	71.88	5.11	13.35	103.13	144.98	0.55	59.83	257.50	0.73
DR	06.12.13	Laguna del Rincon, Dom. Rep.	1117	28.5	8.9	14.78	3.03	−4.05	163.73	7.43	32.90	38.28	218.00	0.50	128.70	189.16	1.14
JM	02.12.13	Parrotee Pond, Jamaica	35,600	32.7	8.6	8.14	2.08	−3.34	6930.00	244.70	860.75	90.41	12,597.00	41.75	1193.00	423.48	1.01

^a^Calcite saturation index: SI_calcite_ = log[(Ca^2+^)(CO_3_^−^)/K_sp_]; K_sp_-solubility product

**Fig. 5:**
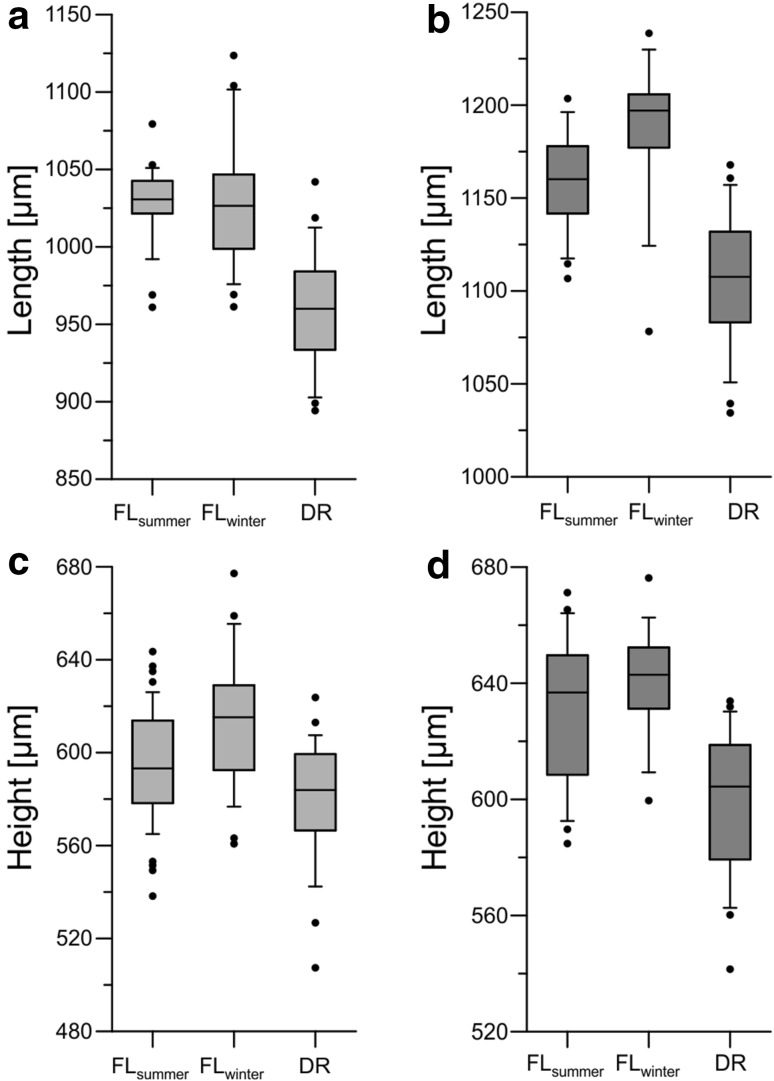
Comparison of length and height measurements of adult *C. salebrosa* between Florida and Dominican Republic: **a** female length, **b** male length, **c** female height, **d** male height; the *middle tick* indicates the median of all values, *grey bars* show 50 % and whiskes 95 % of all values, *black dots* indicate outliers

**Fig. 6:**
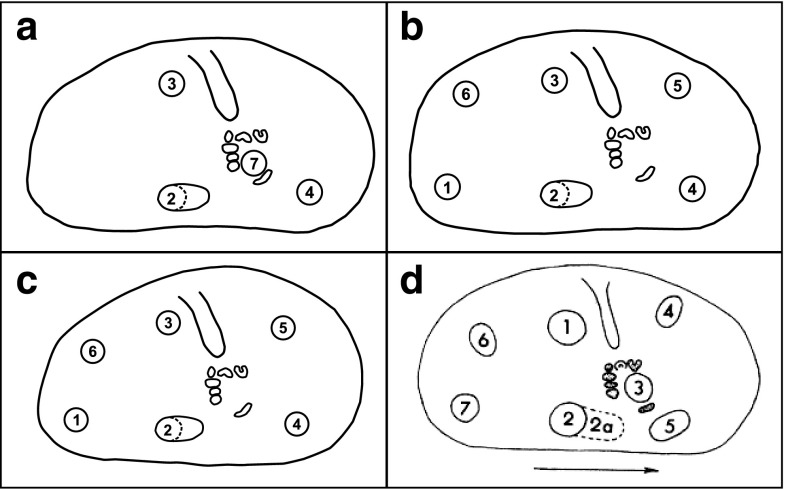
Location of nodes on the valves of *Cyprideis* with numerical designation in this paper: **a** *C. americana* adult, **b** *C. americana* juvenile, **c** *C. salebrosa* adult and juvenile, **d** numerical designation by Sandberg (1964)

**Fig. 7:**
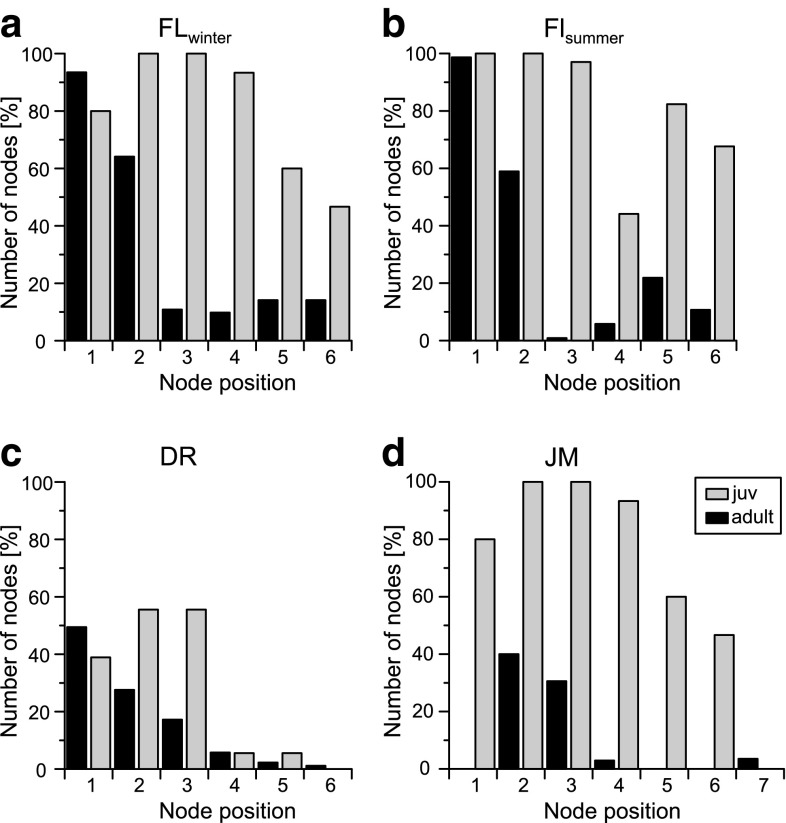
Node frequency of adult individuals (*black bars*) and the last juvenile instar (*grey bars*) of ostracod valves from the investigated sample sites: **a**–**c** *C. salebrosa*; **d** *C. americana*

**Fig. 8:**
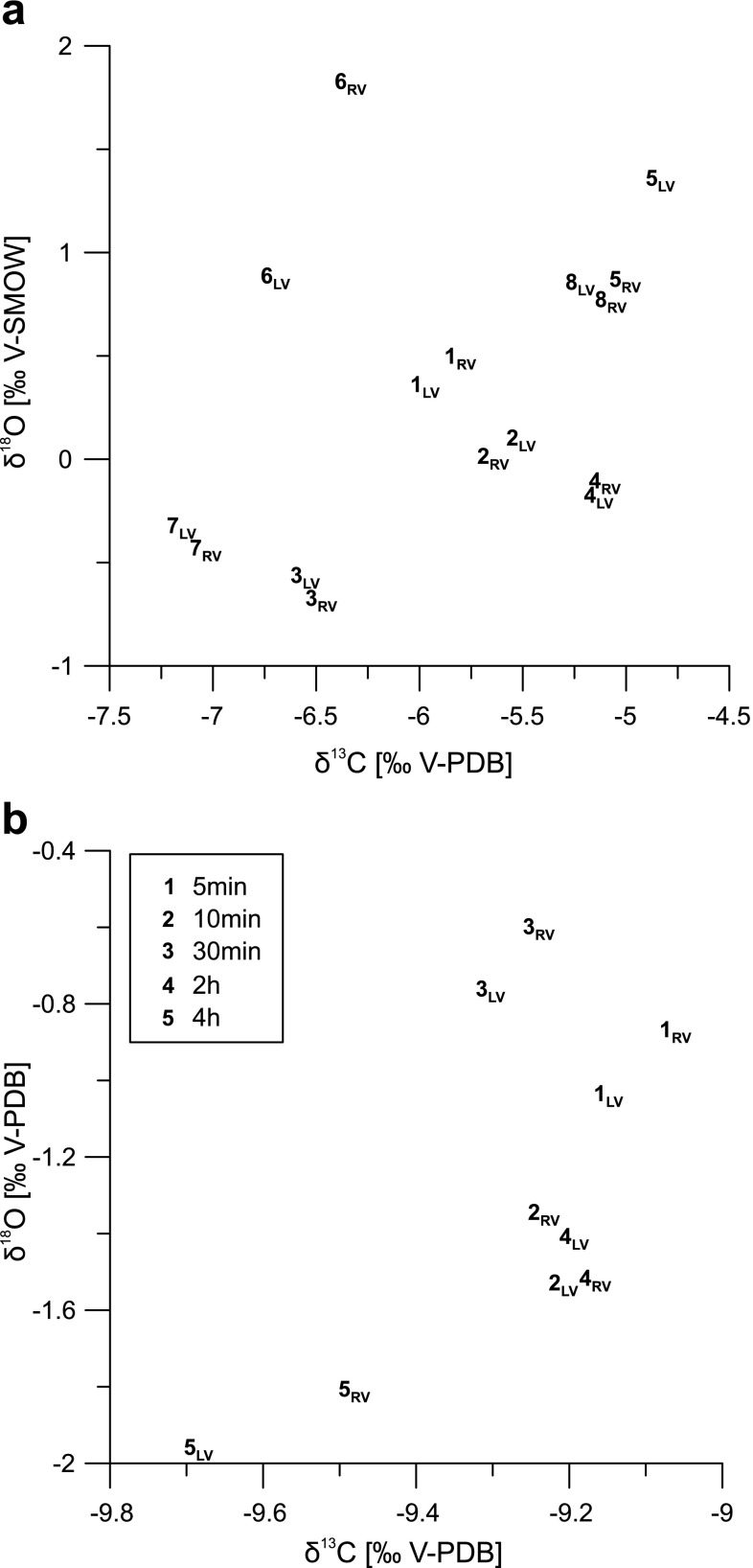
Stable carbon and oxygen isotopes of left and right valves of *C. salebrosa*: **a** comparison of male (1–3) and female shells (4–8) of *C. salebrosa* from DR, **b** different treatment with H_2_O_2_ of right valves of *C. salebrosa* from FL_winter_ (same individuals as in Table 3)

**Fig. 9:**
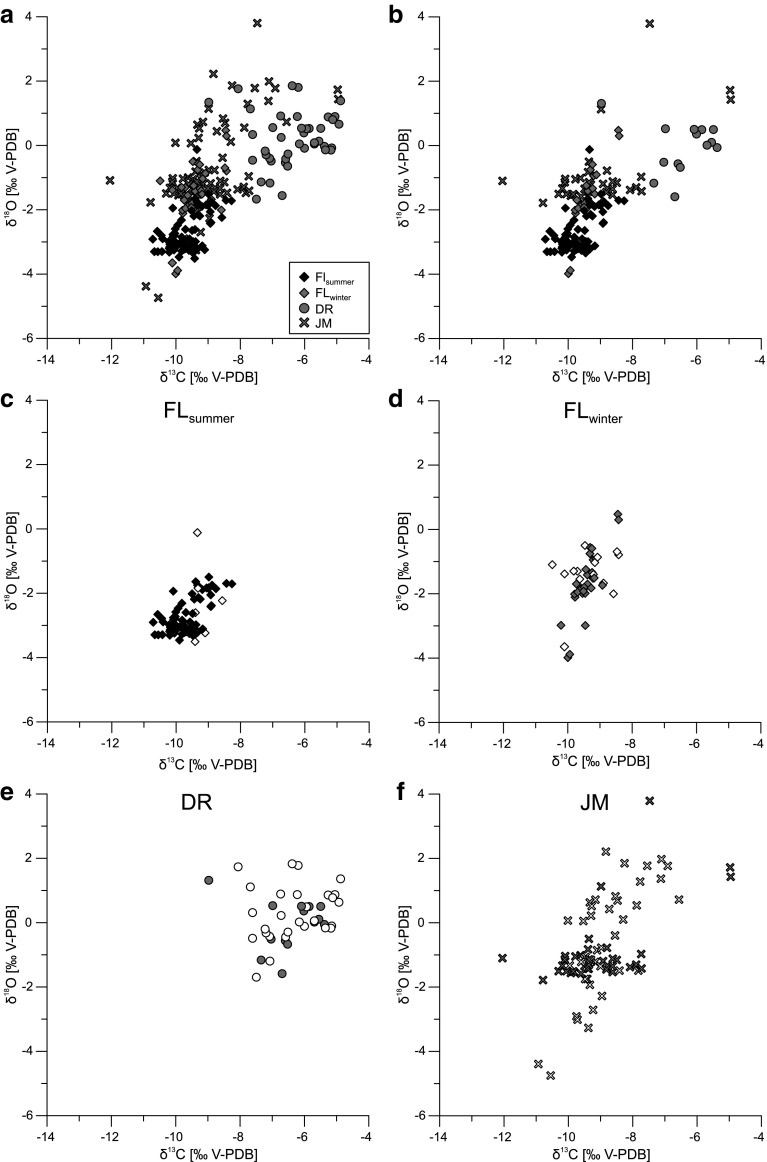
Stable carbon and oxygen isotopes of *C. salebrosa* and *C. americana*: **a** values from all sites, **b** only fresh valves of all sites, **c** FL_summer_, **d** FL_winter_, **e** DR, **f** JM. Data from fresh valves of single sites (**c**–**f**) are shown as *filled symbols* and altered valves as *empty symbols*. *FL*_*summer*_ summer sample Shell Creek, Florida; *FL*_*winter*_ winter sample Shell Creek, Florida; *DR* Laguna del Rincon, Dominican Republic; *JM* Parrotee Pond, Jamaica

**Fig. 10:**
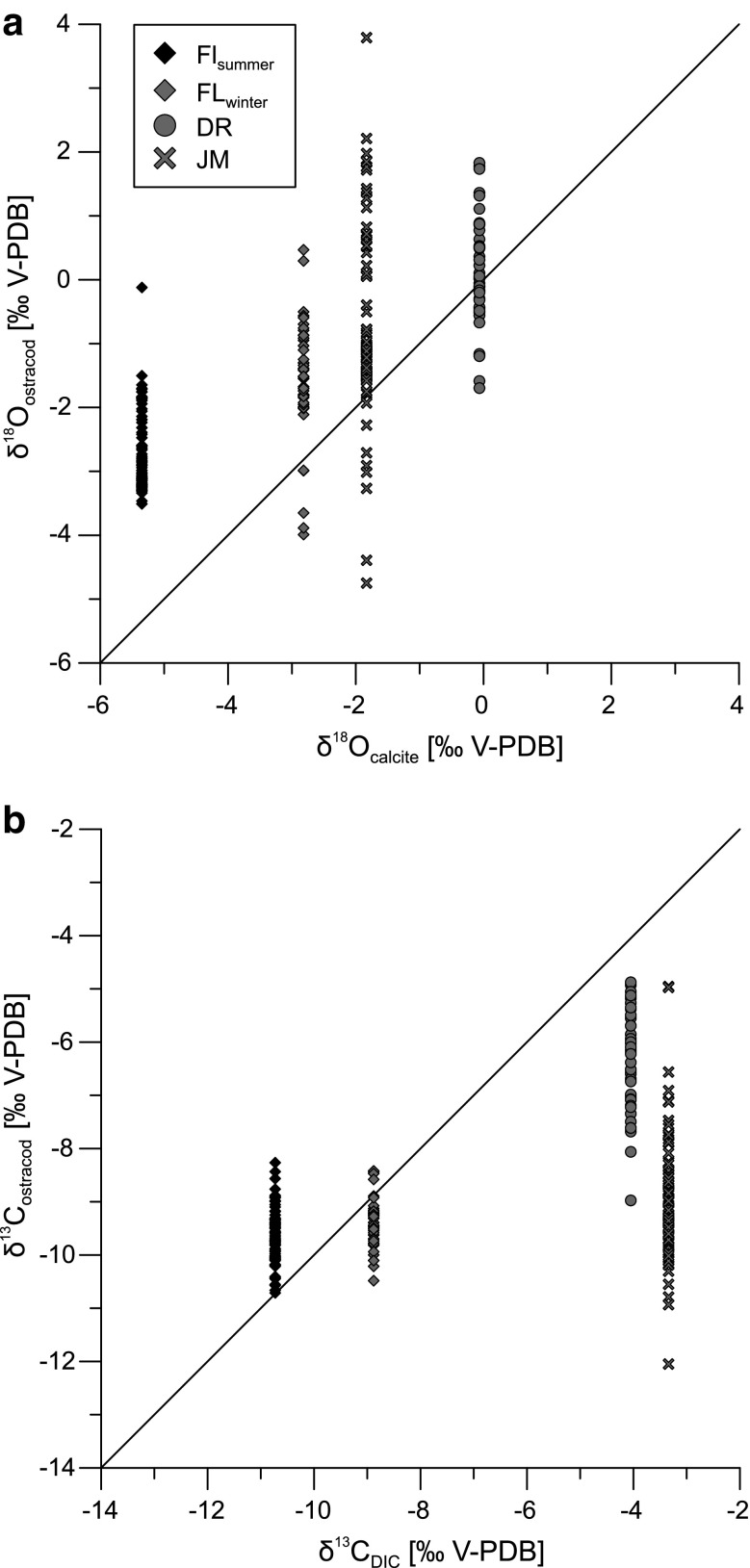
Comparison of the isotopic composition of ostracod valves from *C. salebrosa* and *C. americana* vs. the water in which they evolved: **a** δ^18^O values of ostracod valves vs. δ^18^O values of an abiotic calcite precipitated in the water, **b** δ^13^C values of ostracod valves vs. δ^13^C values of the dissolved inorganic carbon

The chemistry of the two water samples of Shell Creek (Florida) differs slightly between the two seasons. In winter, measured surface water temperature is lower (20.3 °C) and the pH (7.9) and conductivity (938 µS/cm) are higher than in summer (31.2 °C, 7.1, 297 µS/cm). In contrast to the low conductivity in summer, concentrations of bromide, sodium and chloride are higher in summer. In addition, the summer sample is slightly undersaturated in calcite (−0.70), while in winter it is oversaturated (0.73).

The water temperature of Laguna del Rincon (Dominican Republic) is much higher (28.2 °C) than FL_winter_ and is closer to the summer sample of Shell Creek (FL_summer_). The conductivity of Laguna del Rincon is higher than in Shell Creek and ion concentrations are equal or slightly higher, except for calcium and bromide, which are lower, and hydrogen carbonate, which lies in between the summer and winter sample of Shell Creek. Nevertheless, Laguna del Rincon is higher saturated with respect to calcite (1.14) than Shell Creek due to its high pH (8.9).

Parrotee Pond (Jamaica) has the highest ionic concentrations and conductivity (minimum ten times higher) and is the warmest water body (32.7 °C). In addition, it has an equally high pH (8.6) as Laguna del Rincon and is also oversaturated with respect to calcite (1.01).

The overall values of δD and δ^18^O range from −6.0 to 14.78 and −1.74 to 3.03 ‰, respectively. Both samples from Shell Creek have more negative δD and δ^18^O values, whereas the samples from Laguna del Rincon and Parrotee Pond have more enriched values of hydrogen and oxygen isotopes. All water samples deviate negatively from the global meteoric water line (GMWL) (Craig 1961) (Fig. 3). The value of FL_winter_ coincides with the local evaporation line (LEL) reported by Sacks (2002) for West Florida (LEL_Florida_: δD = 4.67(δ^18^O) − 0.21) while FL_summer_ lies above the LEL_Florida_ and closest to the GMWL. The value of Laguna del Rincon plots on a LEL for Lago Enriquillo waters (LEL_DomRep_: δD = 4.7(δ^18^O) + 0.41) (Buck et al. 2005). The value of Parrotee Pond falls on a LEL for Wallywash Great Pond (LEL_Jamaica_: δD = 4.91(δ^18^O) + 0.99) (Holmes et al. 1995).

The overall values of dissolved inorganic carbon (δ^13^C_DIC_) range from −10.73 to −3.34 ‰. The δ^13^C_DIC_ values of Shell Creek are the lowest of the studied sites (FL_winter_: −8.88‰ δ^13^C_DIC_; FL_summer_: −10.73 ‰ δ^13^C_DIC_) and summer values are 1.85 ‰ lower than in winter. The other two sites have also negative δ^13^C_DIC_ values of −3.34 and −4.05 ‰, respectively.

### Ostracod morphology

**Plate 1:**
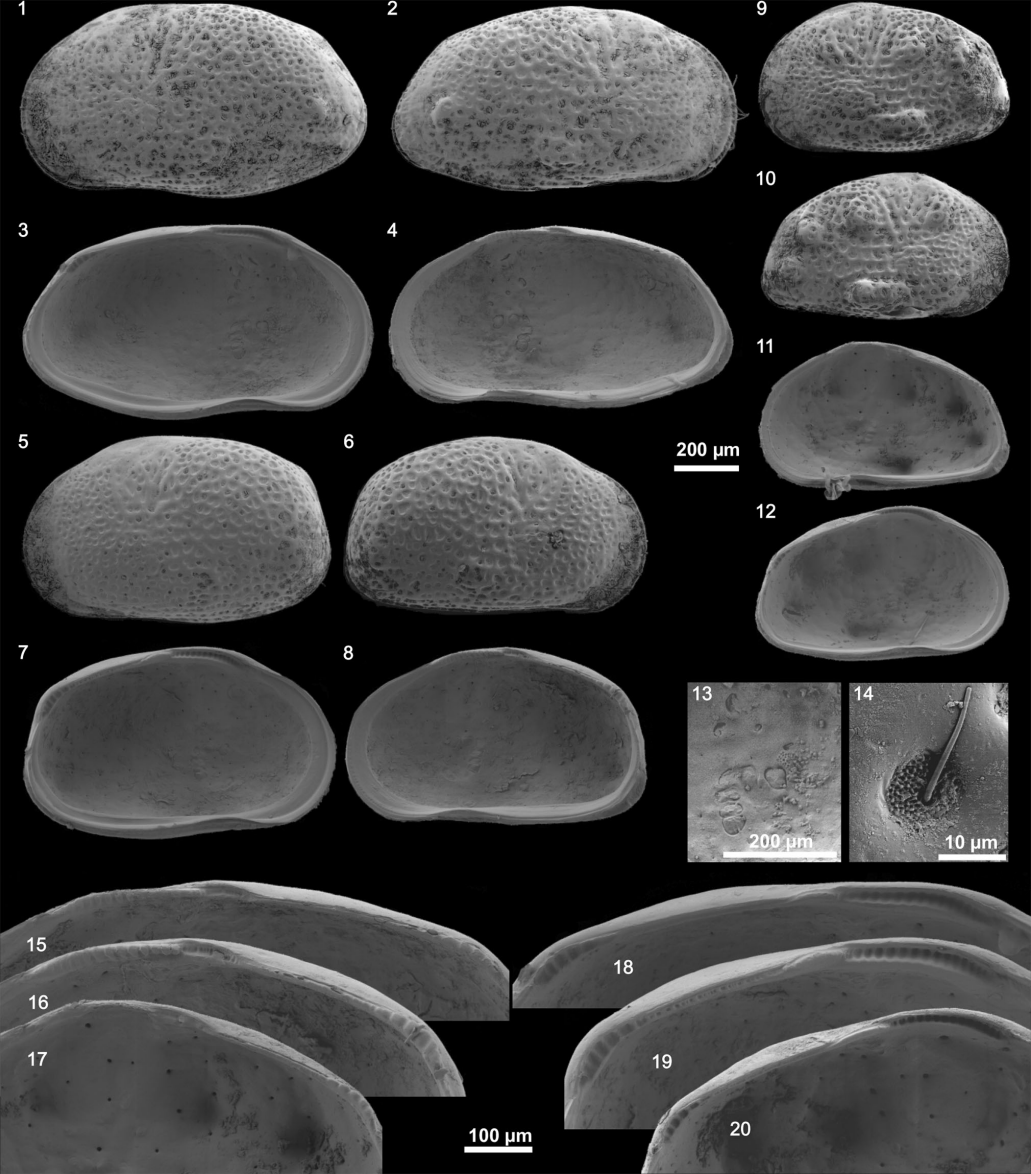
*C. salebrosa* from FL_summer_: (1) Le ♂, (2) Re ♂, (3) Li ♂, (4) Ri ♂, (5) Le ♀, (6) Re ♀, (7) Li ♀, (8) Ri ♀, (9) Le A-1, (10) Re A-1, (11) Ri A-1, (12) Li A-1, (13) muscle scars of Li ♂ (detail of fig. 3), (14) sieve pore of Re ♀, (15) hinge Ri ♂ (detail of fig. 4), (16) hinge Ri ♀ (detail of fig. 8), (17) hinge Ri A-1 (detail of fig. 11), (18) hinge Li ♂ (detail of fig. 3), (19) hinge Li ♀(detail of fig. 7), (20) hinge Li A-1 (detail of fig. 12). *R* right valve, *L* left valve, *e* external view, *i* internal view, *♂* male, *♀* female, *A-1* last juvenile stage)

**Plate 2:**
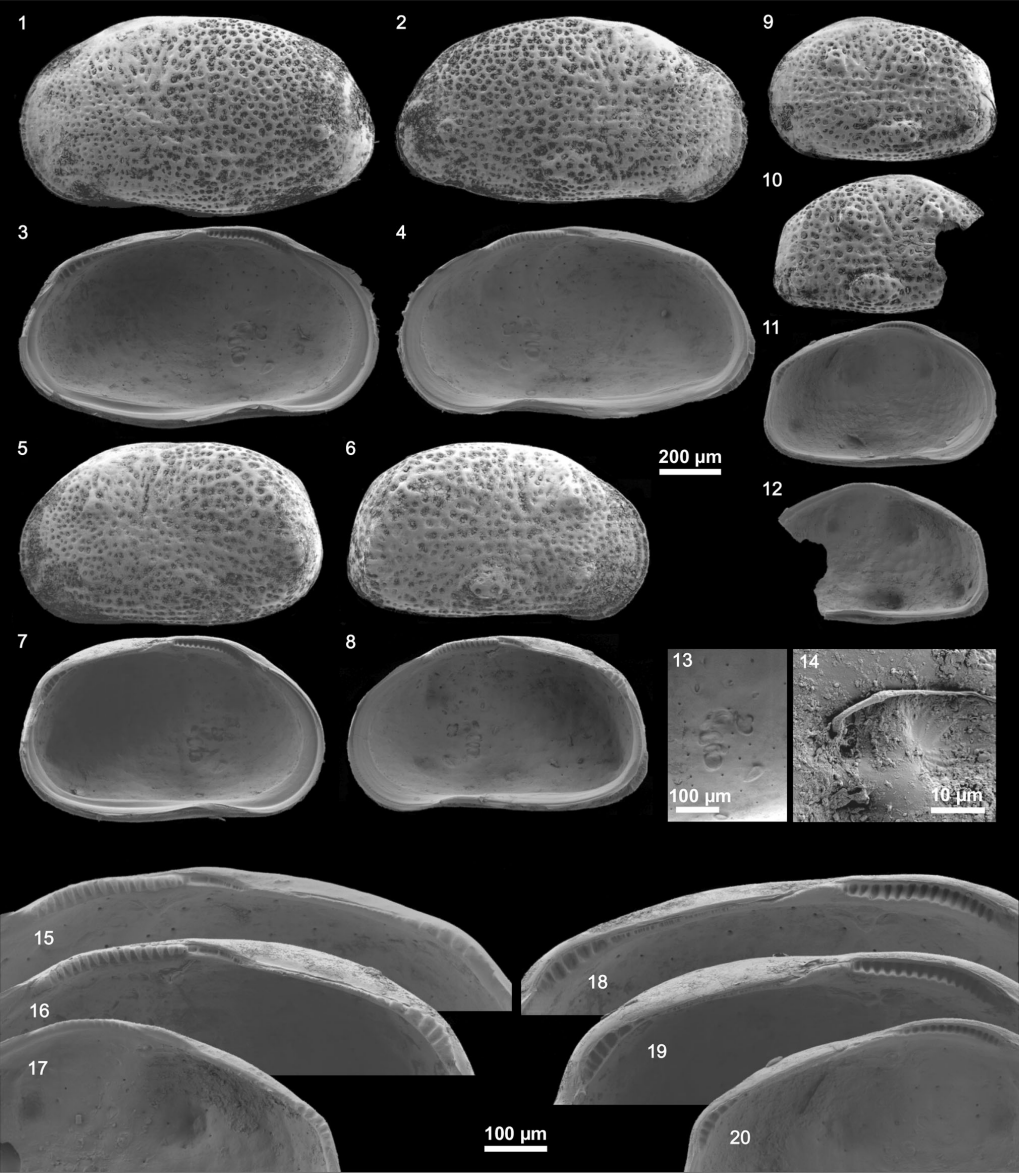
*C. salebrosa* from FL_winter_: (1) Le ♂, (2) Re ♂, (3) Li ♂, (4) Ri ♂, (5) Le ♀, (6) Re ♀, (7) Li ♀, (8) Ri ♀, (9) Le A-1, (10) Re A-1, (11) Li A-1, (12) Ri A-1, (13) muscle scars of Li ♂ (detail of fig. 3), (14) sieve pore of Re ♂, (15) hinge Ri ♂ (detail of fig. 4), (16) hinge Ri ♀ (detail of fig. 8), (17) hinge Ri A-1 (detail of fig. 12), (18) hinge Li ♂ (detail of fig. 3), (19) hinge Li ♀ (detail of fig. 7), (20) hinge Li A-1 (detail of fig. 11). (For abbreviation see plate 1)

**Plate 3:**
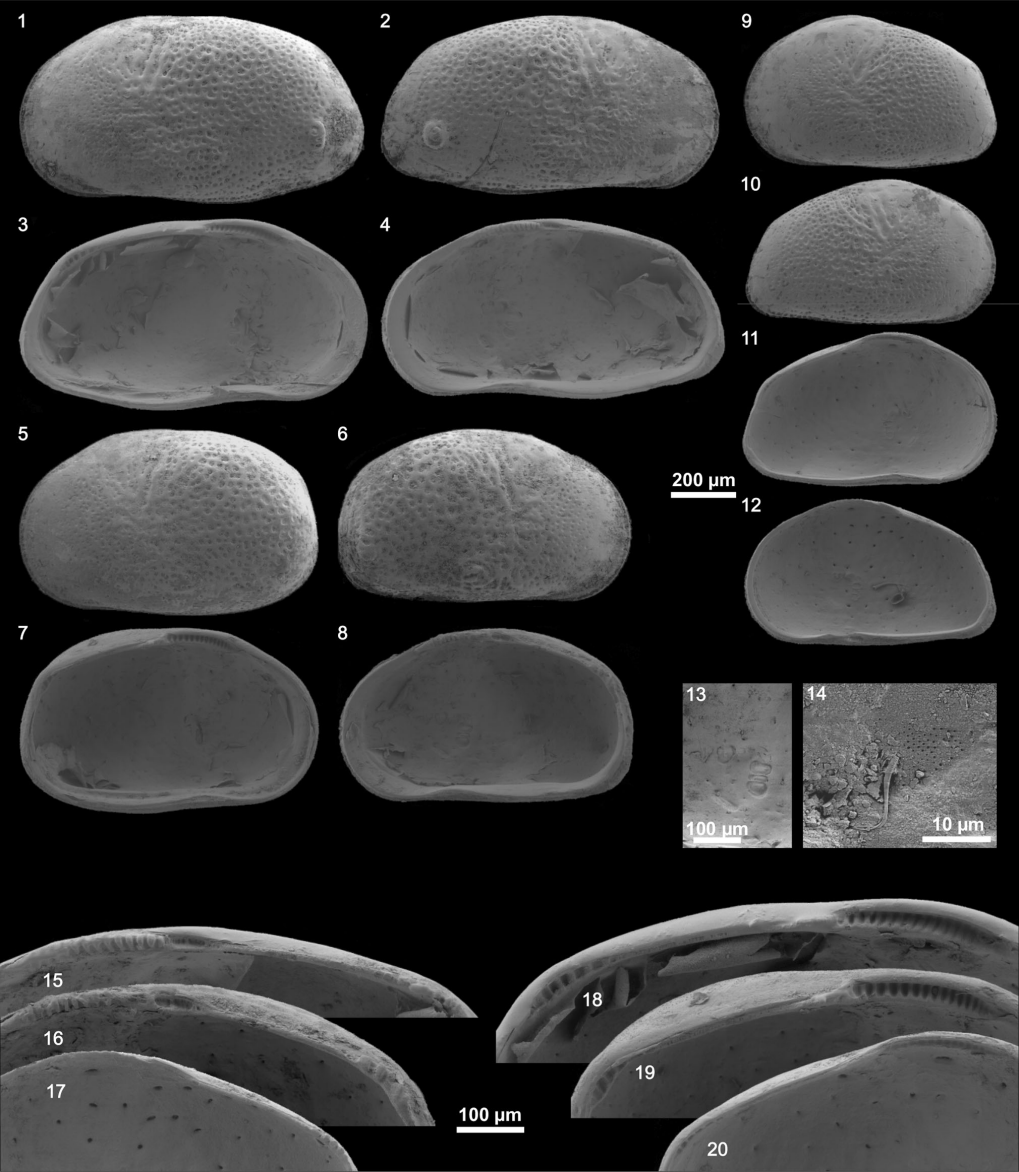
*C. salebrosa* from DR: (1) Le ♂, (2) Re ♂, (3) Li ♂, (4) Ri ♂, (5) Le ♀, (6) Re ♀, (7) Li ♀, (8) Ri ♀, (9) Le A-1, (10) Re A-1, (11) Li A-1, (12) Ri A-1, (13) muscle scars of Ri ♀ (detail of fig. 8), (14) sieve pore of Re ♂, (15) hinge Ri ♂ (detail of fig. 4), (16) hinge Ri ♀ (detail of fig. 8), (17) hinge Ri A-1 (detail of fig. 12), (18) hinge Li ♂ (detail of fig. 3), (19) hinge Li ♀ (detail of fig. 7), (20) hinge Li A-1 (detail of fig. 11). (For abbreviation see plate 1)

**Plate 4:**
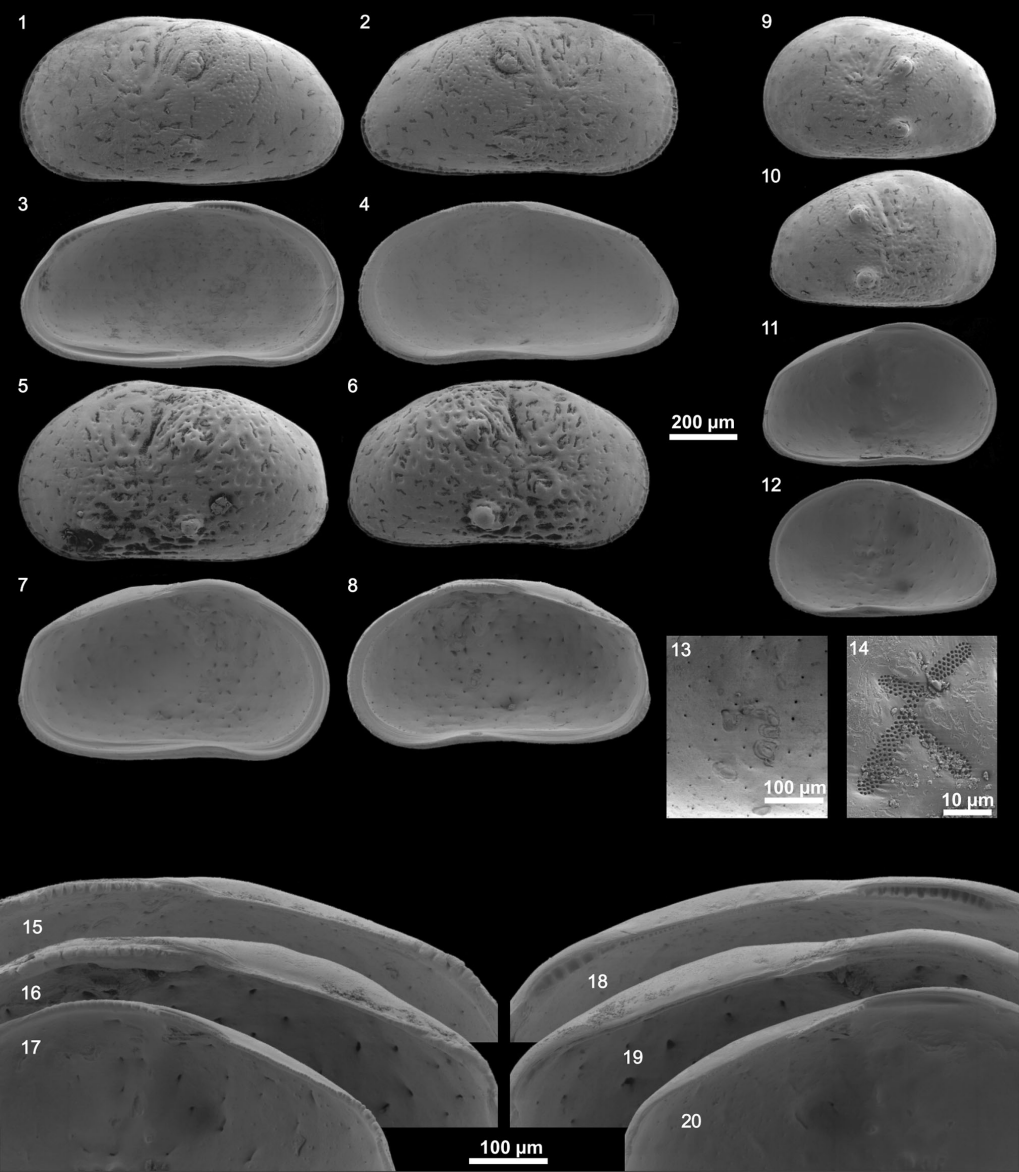
*C. americana* from JM: (1) Le ♂, (2) Re ♂, (3) Li ♂, (4) Ri ♂, (5) Le ♀, (6) Re ♀, (7) Li ♀, (8) Ri ♀, (9) Le A-1, (10) Re A-1, (11) Li A-1, (12) Ri A-1, (13) muscle scars of Ri ♂ (detail of fig. 4), (14) sieve pore of Re ♀, (15) hinge Ri ♂ (detail of fig. 4), (16) hinge Ri ♀ (detail of fig. 8), (17) hinge Ri A-1 (detail of fig. 12), (18) hinge Li ♂ (detail of fig. 3), (19) hinge Li ♀ (detail of fig. 7), (20) hinge Li A-1 (detail of fig. 11). (For abbreviation see plate 1)

We identified two species of the genus *Cyprideis* (Plates 1, 2, 3, 4). Within the samples from Laguna del Rincon and Shell Creek we found individuals of *C. salebrosa*, whereas *C. americana* was only found in Parrotee Pond.

Surface ornamentation differed from smooth to strongly pitted within one species at a single location. *C. americana* has well developed sieve pores with great differences in shape (rounded to elongated and branched) and size while *C. salebrosa* has just in some cases small rounded sieve pores (Plate 1–4, fig. 14 each).

#### Size measurements

Overall, *C. salebrosa* is larger than *C. americana* (Table 2). In both species males are longer than females (Fig. 5) and left valves of the same individual are larger than the right valves (Table 2). Within both species the length/height proportion of the juveniles show the same trend as the females.

**Table 2:** Mean, minimal, maximal and standard deviation of size measurements of male, female and juvenile *Cyprideis*

Sample	Valve	No. of valves	Length				Height			
			Mean	Min	Max	SD	Mean	Min	Max	SD
FL_summer_	Male	71	1155	1085	1204	±24	619	563	671	±25
*C. salebrosa*	Female	93	1016	950	1079	±25	595	538	644	±22
	A-1	24	815	770	858	±33	480	494	488	±12
	A-2	0	–	–	–	–	–	–	–	–
	A-3	0	–	–	–	–	–	–	–	–
FL_winter_	Male	27	1190	1078	1239	±33	641	600	676	±16
*C. salebrosa*	Female	42	1029	961	1123	±38	612	561	677	±27
	A-1	17	836	746	944	±44	486	450	521	±20
	A-2	3	621	591	642	±22	376	255	387	±10
	A-3	1	481	–	–	–	255	–	–	–
DR	Male	44	1107	1034	1167	±39	600	542	634	±25
*C. salebrosa*	Female	42	961	893	1042	±36	581	507	624	±24
	A-1	12	769	708	819	±41	464	443	489	±14
	A-2	3	556	524	591	±28	353	343	369	±12
	A-3	0	–	–	–	–	–	–	–	–
JM	Male	19	1064	1008	1120	±33	543	499	590	±27
*C. americana*	Female	39	970	894	1065	±45	563	517	623	±27
	A-1	8	760	667	889	±74	443	551	409	±32
	A-2	0	–	–	–	–	–	–	–	–
	A-3	0	–	–	–	–	–	–	–	–

Male *C. salebrosa* have an overall length ranging from 1034 to 1239 µm and height ranging from 542 to 676 µm, whereas the length of male *C. americana* ranges between 1008 and 1120 µm and the height between 498 and 589 µm. Females have a length ranging between 894 and 1124 µm and a height of 507–677 µm for *C. salebrosa* and a length ranging from 894 to 1065 µm and a height ranging from 500 to 623 µm for *C. americana*. Adult and juvenile individuals of *C. salebrosa* from Shell Creek are bigger than those from Laguna del Rincon. In addition, specimens from FL_winter_ are larger than from FL_summer_ (Table 2; Fig. 5).

#### Node formation

Nodes (“tubercles”, hollow protuberances) occur on the valves of *C. salebrosa* and *C. americana* at seven positions (Fig. 6). Deviating from Sandberg we numbered these nodes according to their frequency in *C. salebrosa* from the Dominican Republic (position 7 occurs in *C. americana* only). Adult *C. salebrosa* form nodes at six of the seven positions (1–6), whereas adult *C. americana* builds nodes at positions 2–4 and 7. Both species show enhanced node formation on right valves (Table 3). Juvenile specimens develop nodes in a much higher frequency than adults (Fig. 7). Dissimilar to adult *C. americana*, juveniles of this species form nodes only at positions 1–6.

**Table 3:** Relative frequency of nodes on *Cyprideis* (mean values per node)

Sample	Valve	Number of valves	Frequency (%)						
			0 nodes	1 node	2 nodes	3 nodes	4 nodes	5 nodes	6 nodes
FL_summer_	Male RV	53		3.8	58.5	26.4	5.7	3.8	1.9
*C. salebrosa*	Male LV	53	3.8	62.3	22.6	11.3			
	Female RV	59		1.7	52.5	32.2	13.6		
	Female LV	59	1.7	64.4	30.5	3.4			
	juv. (A-1) RV	17				11.8	11.8	23.5	52.9
	juv. (A-1) LV	17				23.5	17.7	29.4	29.4
FL_winter_	Male RV	20		5.0	40.0	20.0	20.0	5.0	10.0
*C. salebrosa*	Male LV	22	9.1	45.5	27.3	4.6	9.1		4.6
	Female RV	25		4.0	68.0	12.0	12.0	4.0	
	Female LV	25	8.0	72.0	16.0	4.0			
	juv. (A-1) RV	9					44.4		55.6
	juv. (A-1) LV	6			16.7	16.7	16.7	16.7	33.3
DR	Male RV	27	48.2	33.3	3.7	14.8			
*C. salebrosa*	Male LV	27	70.4	14.8	3.7	7.4	3.7		
	Female RV	16	31.3	25.0	18.8	12.5	6.3	6.3	
	Female LV	17	35.3	17.7	29.4		11.8	5.9	
	juv. (A-1) RV	7	14.3	28.6	28.6	14.3		14.3	
	juv. (A-1) LV	11	45.5		27.3	27.3			
JM	Male RV	46	54.4	23.9	19.6	2.2			
*C. americana*	Male LV	45	71.1	13.3	13.3	2.2			
	Female RV	39	33.3	25.6	30.8	10.3			
	Female LV	40	52.5	22.5	17.5	7.5			
	juv. (A-1) RV	9			100.0				
	juv. (A-1) LV	2			100.0				

The number of investigated individuals range from 20 to 59 for adults (2-17 for instar A-1). Frequencies with value zero are left out

Adults of *C. salebrosa* form most nodes at position 1 on both valves. The relative frequency of node 2 is also increased on the Floridian *Cyprideis*, but in a minor frequency than the first. The other nodes are developed in a lower frequency. The Dominican specimens form nodes in a lower percentage (~53 %) than the Floridian (~96 % FL_winter_ and 99 % FL_summer_). They show a decreasing frequency from node 1–6. Juvenile individuals from Florida develop nodes at all six positions in a high frequency whereas the Dominican juvenile specimens grow nodes less often and mostly on position 1–3.

Adult individuals of *C. americana* have most nodes on position 2 followed by position 3. Nodes on position number 4 and 7 are the fewest. The A-1 juvenile instar forms nodes only on position 1–6 but in a much higher frequency than the adults.

### Stable isotopes of ostracod valves

The comparison of left and right valves of the same individuals of *C. salebrosa* (Table 4; Fig. 8a) shows slight differences in the isotopic composition (up to 0.94 ‰ for δ^18^O and 0.36 ‰ for δ^13^C) that exceed the instrumental precision. However, the deviation is comparatively small (*R* ^2^ = 0.75 for δ^18^O and *R* ^2^ = 0.97 for δ^13^C) to the whole range of isotopic values between the individuals from a single locality (up to 2.50 ‰ for δ^18^O and 2.32 ‰ for δ^13^C).

**Table 4:** Differences in the stable isotope values of left and right valves of *C. salebrosa*

Sample	Shell	Gender	Preservation	Treatment with H_2_O_2_	Difference in δ^18^O [‰ V-PDB]	Difference in δ^13^C [‰ V-PDB]
DR	1	♂	Neutral	–	0.14	0.17
	2	♂	Neutral	–	0.09	0.14
	3	♂	Neutral	–	0.11	0.07
	4	♀	Neutral	–	0.07	0.02
	5	♀	Altered	–	0.49	0.18
	6	♀	Altered	–	0.94	0.36
	7	♀	Neutral	–	0.11	0.11
	8	♀	Neutral	–	0.08	0.14
FL_winter_	1	♀	Neutral	RV 5 min	0.17	0.09
	2	♀	Fresh	RV 10 min	0.18	0.03
	3	♀	Fresh	RV 30 min	0.16	0.06
	4	♀	Neutral	RV 2 h	0.11	0.03
	5	♀	Fresh	RV 4 h	0.15	0.20
					Ø 0.22	Ø 0.12

For preservation see text and Fig. 9

*DR* difference between male and female valves, *FL*_*winter*_ different H_2_O_2_ treatments of right valves (RV)

Valves treated with hydrogen peroxide differed from untreated valves of the same individuals up to 0.18 ‰ for δ^18^O and 0.20 ‰ for δ^13^C (Table 4; Fig. 8b). This deviation is as low as between untreated valves (*R* ^2^ = 0.93 for δ^18^O and *R* ^2^ = 0.91 for δ^13^C), even after 4 h of treatment with hydrogen peroxide. However, H_2_O_2_ was only used for cleaning if absolutely necessary for subsequent isotopic measurements.

Overall oxygen and carbon isotopic values of all ostracod valves vary from −4.75 to 3.79 ‰ and from −12.05 to −4.88 ‰, respectively (Table 5; Fig. 9).

**Table 5:** Range of isotopic measurements of ostracod valves in respect to male, female and juvenile (A-1) and calculated mean fractionation factor (α_calcite-water_)

Sample	Number of measurements				Stable isotopes		
	♂	♀	A-1	Total	δ^18^O [‰ V-PDB]	δ^13^C [‰ V-PDB]	α_(calcite-water)_
FL_summer_	43 (38)	28 (27)	8 (8)	79 (73)	−3.51 to −0.12 (−3.46 to −1.50)	−10.71 to −8.26 (−10.71 to −8.26)	(1.0299 ± 0.56)
FL_winter_	17 (12)	26 (15)	2 (2)	45 (29)	−3.99 to 0.47 (−3.99 to 0.47)	−10.48 to −8.42 (−10.21 to −8.42)	(1.0306 ± 0.99)
DR	19 (10)	19 (3)	4 (1)	42 (14)	−1.70 to 1.83 (−1.59 to 1.32)	−8.97 to −4.88 (−8.97 to −5.38)	(1.0277 ± 0.77)
JM	41 (26)	37 (12)	1 (0)	79 (38)	−4.75 to 3.79 (−1.78 to 3.79)	−12.05 to −4.96 (−12.05 to −4.96)	(1.0278 ± 1.11)

Values in brackets from fresh ostracods only

However, the isotopic values differ between sites. The isotopic range for *C. salebrosa* from Shell Creek is the lowest and similar between summer and winter with δ^18^O values from −3.51 to −0.12 ‰ and from −3.99 to 0.47 ‰ and δ^13^C values from −10.71 to −8.26 ‰ and −10.48 to −8.42 ‰. The δ^18^O values are lower in summer while the δ^13^C values are nearly the same. The isotopic variability for *C. salebrosa* from Laguna del Rincon is higher than in specimens from Shell Creek, ranging from −1.70 to 1.83 ‰ for δ^18^O and −8.97 to −4.88 ‰ for δ^13^C. The isotopic range for *C. americana* is nearly as high as the overall range of all ostracod values for both δ^18^O and δ^13^C. For isotopic values of ‘fresh’ ostracods, the range for oxygen isotopes gets smaller, especially for *C. americana*, whereas the range of carbon isotopes remains nearly the same. Isotopic values of ostracod valves differ from the values of an abiotically formed calcite in their host water (Fig. 10). For δ^18^O values, the ostracods have a positive offset to their host water, while δ^13^C values are negative except for FL_summer_.

Investigations of the genders of *Cyprideis* in correlation with the isotopic results showed no significant difference between male and female specimens (Table 4). Juveniles from Laguna del Rincon used to have the lowest carbon isotopes and juveniles of FL_summer_ have the lowest oxygen isotopic values.

The preservation of the valves did not give a clear result on the isotopic pattern. ‘Altered’ valves of Jamaican *Cyprideis* and from FL_summer_ seem to have a higher variation in oxygen isotopes, whereas DR and FL_summer_ valves show no distinct pattern (Fig. 9b–f).

## Discussion

### Physico-chemical characteristics of the studied sites

The in situ measurements provide basic information on the different (hydrological) characteristics of the study sites. Which, in turn, have varying isotopic and ionic composition during the year.

Measured water temperature and electrical conductivity (EC) values of Shell Creek are typical for the stream and correspond well with measurements of the USGS sample station (U.S. Geological Survey 2014) in both months. The temperature and conductivity data of the USGS water station at Shell Creek (02297635) provide information representative for annual conditions. These data display a correspondence of stream temperature and annual air temperatures of Florida.

The measured winter temperatures of Laguna del Rincon and Parrotee Pond match with the temperature of Shell Creek in summer (Table 3). That could be explained by a generally warmer climate in the Dominican Republic and Jamaica. Information on annual environmental changes of Laguna del Rincon and Parrotee Pond are not available. However, annual air temperature variation is similar at all sites, but daily heating of the water body may be much higher in lentic waters, where water movement and continuous mixing is missing.

Conductivity of Shell Creek is mainly influenced by the wet season from May to October resulting in higher runoff during that time and a depletion of chemical components. Shell Creek exhibits, however, freshwater conditions during the whole year. Higher salinity of Shell Creek can only be caused by entering of seawater during spring tides. Considering conductivity values of the sampling years, Shell Creek was not affected by such an event (U.S. Geological Survey 2014).

Laguna del Rincon has an ionic composition similar to FL_winter_ indicating freshwater conditions (Table 1). It is most likely, that changes in the proportion of evaporation and precipitation changes the ion content of the lake the most. The ionic concentration of Parrotee Pond is much higher than at the other sites and can be explained by mixing with marine water due to its proximity to the coast. In particular, Bromine as a tracer for saltwater mixing is enriched in Parrotee Pond (Fig. 2). Its ionic concentration, however, is still lower than seawater which is probably caused by the income of freshwater from precipitation in the catchment area.

Concerning the isotopic composition, the close position of δD and δ^18^O to the GMWL of Shell Creek samples indicate a low influence on the stream by evaporation (Fig. 3). The low stream velocity during low runoff in winter can also cause the accumulation of heavier isotopes (Table 1; Figs. 2, 3, 4) and explain slightly higher isotopic values of the winter sample. Changes in the isotopic composition of streams (δD and δ^18^O) reflect recent rainfall while accumulation of water in lakes buffers the precipitation influence (e.g., Leng and Marshall 2004). Price et al. (2008) investigated fluctuations of isotopic signatures from precipitation in south Florida and found very low δD and δ^18^O values prior to the passage of hurricanes and during cold fronts with an oceanic vapor source in the west of Florida. This depletion is independent from the amount of precipitation and changes mainly with the vapor source. This seasonal variation will also affect Shell Creek but fluctuation may be buffered by the inflow of groundwater.

Both, Laguna del Rincon and Parrotee Pond are much more enriched in heavy isotopes (D and ^18^O) than Shell Creek (Figs. 3, 4). The enrichment of δD and δ^18^O along LELs indicate the enrichment through evaporation (Fig. 3). Therefore, changes in the proportion of precipitation and evaporation (P/E) have to be considered to influence both locations. Similar values to Parrotee Pond (δ^18^O and δ^13^C_DIC_) have been reported from Wallywash Great Pond located in the east of the pond (Holmes et al. 1995). This lake is mainly influenced by evaporation. But, saltwater mixing may also influence the isotopic composition of Parrotee Pond periodically.

The stream flow of Shell Creek provokes a permanent inflow of fresh water and outflow of older water and, thus, prevents the accumulation of ^13^C by biological activity or CO_2_ exchange with the atmosphere (Atekwana and Krishnamurthy 1998). The exchange with atmospheric CO_2_ over time increases the δ^13^C_DIC_ of lakes and may explain higher values in DR and JM (−4.05 and −3.34 ‰). But, isotopic equilibrium with atmospheric CO_2_ would lead to values up to +3 ‰ and both water bodies are probably not in equilibrium with the atmosphere (Leng and Marshall 2004). Additionally, photosynthetic activity may cause high fluctuations of ^13^C in Laguna del Rincon and Parrotee Pond.

### Ecological and morphological variability of *Cyprideis*

We found *C. salebrosa* and *C. americana* at sites within their reported geographical range*. C. salebrosa* is widely distributed along the American continent. It occurs mainly at locations around the margin of the Gulf of Mexico and islands of the West Indies, but also at La Plata (Argentina), Venezuela and Trinidad, and at single locations in Kansas and Central Missouri (e.g., Sandberg 1964; Garbett and Maddocks 1979; Keyser 1978; Stout 1981). Sharpe (1908) reported the occurrence of *C. americana* from Jamaica Bay and Brighton Beach, New York. Further investigations on *C. americana* are restricted to saline and hypersaline lakes of the Bahamas (e.g., Teeter et al. 1987a, b; Teeter and Quick 1990; Bowles 2013).

A main factor controlling the distribution of ostracods is salinity (e.g., Puri 1966; Kilenyi 1969; Horne and Boomer 2000). *Cyprideis* is known to flourish in brackish habitats, but species have different tolerances to salinity (Sandberg 1964). Keyser (1978) found populations of *C. salebrosa* in environments with salinities between 0.5 and 4.5 psu. Garbett and Maddocks (1979) found *C. salebrosa* all over the Bays of Texas in areas of mixing water bodies, where streams enter the bays or where rivers enter the Gulf. *C. americana* occurs only in waters with salinities higher than 10 psu. More detailed studies on the ecology of *C. salebrosa* and *C. americana* are missing. However, our observations are in accordance with previous findings on *C. salebrosa* and *C. americana.* Their occurrence is, thus, predominantly related to different salinities.

Besides that, both species show morphological variability that can be addressed to local and seasonal differences. For instance, the presence of large irregular sieve pores on *C. americana* (Plate 4, fig. 14) may reflect a high salinity environment but seasonal changes may increase the variation of their shape depending on the conditions during valve formation (e.g., Rosenfeld and Vesper 1978; Rosenfeld 1982; Medley et al. 2008; Pint et al. 2012; Bowles 2013).

#### Size variability of *C. salebrosa*

The valve size of *C. salebrosa* differs between the investigated sites (Table 2; Fig. 5). Temperature differences at the sites and between seasons seem to be a reasonable explanation for these differences.

Annual temperature of the Dominican Republic is generally higher than in Florida and the Dominican specimens are smaller than the ones from Florida.

In addition, the largest specimens occur in the Floridian winter sample where temperature is lower than during summer. This temperature-size relationship can be observed in both sexes (Fig. 5) and the last two juvenile stages (Table 2). Considering the restricted data set, this impact of temperature on valve size is a hypothesis that needs to be tested in further studies.

However, this reverse temperature-size dependency of *C. salebrosa* was already observed by Schweitzer and Lohmann (1990) who investigated the life-history of *Cyprideis* species and their ontogenetic. Schweitzer and Lohmann (1990) addressed the seasonal size variation of *C. salebrosa* to the life cycle of the species. In *C. salebrosa* two peaks of juvenile abundance were observed in early June and August (Schweitzer and Lohmann 1990). The first group matures very fast over summer and the second progresses towards adulthood much slower during late autumn and winter. Consequently, *C. salebrosa* has a longer intermolt period during lower temperatures and the animal has more time to grow until the next molting, which leads to larger individuals during the cold season. Marco-Barba (2010) found a similar population dynamic for *Cyprideis torosa* on the Iberian Peninsula with high abundances of adult individuals in May or June and in September to January at different sites indicating two similar maturation cycles as in *C. salebrosa*. A comparison of *C. salebrosa* specimens from Massachusetts with other locations showed also that specimens from more southern regions are smaller (Schweitzer and Lohmann 1990). These authors found regional temperature differences to be slightly larger than seasonal fluctuations.

An inverse temperature correlation has also been observed in other ostracod species (Martens 1985; Cronin 2005). Martens (1985) investigated the growth of *Mytilocypris henricae* under defined temperature conditions and observed smaller individuals and a faster molting rate at higher temperatures in the range of 15–25 °C. In the contrary, individuals grown at 10 °C grew slowest and were the smallest. Also Cronin et al. (2005) found individuals of *Loxoconcha matagordensis* from natural environments to be smaller (seasonally and regionally) at higher temperature. They assumed two possible reasons for the size differences: (a) slower carapace growth at low temperatures due to reduced metabolic activity during the molting process; (b) *L. matagordensis* populations with small-shelled individuals in summer produce a high number of small eggs that have a selective advantage to survive until spring breeding in a reduced *Zostera* seagrass habitat during winter. A temperature dependency seems to be very reasonable but since the number of sites and knowledge on environmental requirements of *C. salebrosa* is small this relation may not causal and other parameters could be responsible for that size differences.

Frenzel and Boomer (2005) summarized possible influences of salinity and parameters connected to salinity on the valve size of brackish ostracods. Positive (Hartmann 1963), negative (Barker 1963; Van Harten 1975; Martens 1985) as well as no correlation (Kilenyi 1971; Vesper 1972a; Frenzel 1991) between valve size and salinity have been recognized within several freshwater and marine ostracod species at different salinity ranges. Changes from the salinity optimum of a species may reduce the size of ostracod individuals in both directions (Keen 1982; Neale 1988). This phenomenon was observed in *Cyprideis torosa* (Van Harten 1996; Boomer and Frenzel 2011, Boomer et al. 2016) at salinities above and below 8–9 psu. This size reduction was explained with the change between hypo- and hyper-osmoregulation. Additionally, some authors suggested other parameters partly connected to salinity changes like food supply and population density (Puri 1966; Keen 1971; Vesper 1972a) or calcification (Kühl 1980) to influence valve size. The connection between changes in salinity and other factors on valve size is, however, still not fully understood.

The small measured salinity range and the missing knowledge on seasonal salinity changes and the osmotic optimum of *C. salebrosa* proscribe a direct correlation using the limited number of samples.

#### Variability in noding

Former studies on the nodocity of *Cyprideis* species showed that the location of the nodes is anatomically determined (Sandberg 1964; Vesper 1972b; Van Harten 1975, 1996, 2000; Frenzel 1991; Keyser 2005) and restricted to seven possible positions. The combination of their positions at the valve varies between species (Sandberg 1964). We found nodes on positions 1–6 and 1–4 + 7 on valves of adult *C. salebrosa* and *C. americana* (Fig. 6). Noding on *C. salebrosa* corresponds with findings of Sandberg (1964). A description on the node position of *C. americana* is not known so far. It differs from other *Cyprideis* species but their occurrence is in accordance with the proposed seven potential positions of Sandberg (1964). Interestingly, the node position of juvenile *C. americana* (1–6) differs from adult specimens and is the same as in juvenile and adult *C. salebrosa* (Fig. 6). A change in the combination of the positioning of nodes between juvenile and adult individuals has not been observed before at a *Cyprideis* species.

Noding is a problem of osmoregulation capacities during molting in low saline waters (Keyser and Aladin 2004; Keyser 2005). Consequently, varying noding frequency of the same species indicates salinity differences at the time of their valve formation. Accepting this theory the lower frequency of nodes of the Dominican specimen (adult and juvenile) would indicate higher salinity conditions in Laguna del Rincon than in Shell Creek while differences in seasonal conditions of the stream cannot be observed (Fig. 7). Although, higher salinity is not observed in the water measurements, higher salinity values during an earlier season is possible.

Although, the salinity-nodocity relationship is linear in the laboratory (Frenzel et al. 2012), an additional factor influences the node formation under natural conditions (Van Harten 2000).

The calcium concentration in the water seems to be an additional factor, since dissolved Ca^2+^ ions are needed for osmoregulation (Keyser 2005; Frenzel et al. 2012). Van Harten (2000) suggested the amount of CO_2_ influencing the pH value as an additional factor. The pH value is connected with the CaCO_3_ solubility and with the dissolved Ca^2+^ in the water. Much higher pH values and higher calcite saturation in Laguna del Rincon and Parrotee Pond would support the hypothesis of Van Harten (2000) but not the amount of dissolved Ca^2+^ (Table 1). However, a minor influence of these factors on the nodocity cannot be excluded.

Furthermore, we found stronger noding of the last juvenile stage of both species (Fig. 7). The phenomenon of stronger noding in the juvenile stages of *Cyprideis* has already been noticed by other authors (e.g., Sandberg 1964; Vesper 1972b; Frenzel 1991; Frenzel et al. 2012). Frenzel et al. (2012) suggest a higher surface to volume ratio, enabling a higher water inflow or their non-mature osmoregulation capacities to cause stronger noding.

More frequent noding was also observed on right valves of both species (Table 3). The asymmetrical appearance of nodes on the valves of *C. torosa* was explained by Keyser and Aladin (2004) as a result of the behavior of the animal laying on the side of the shell during molting. In this case, animals have to lie preferentially on the left side of the shell to explain the higher frequency of nodes on the right valve. Possibly an anatomical asymmetry of the animal could be a reason for that. In fact, the left valve is bigger than the right one and a higher weight could be accepted.

### Intra-individual stable isotope variability

The composition of two valves of a single carapace is statistically inseparable (e.g., Heaton et al. 1995; Keatings et al. 2006). But, dissolution of older valves or the pre-treatment of valves before measurement may alter the isotopic composition of the shell. Thus, possible effects have to be examined. Male and female individuals showed a similar low deviation between their valves (Table 4; Fig. 8a). Only one altered female showed a significant higher deviation of 0.94 and 0.36 ‰ for δ^18^O and δ^13^C, respectively. Therefore, alteration has to be taken into account to change within sample variability.

Further, pre-treatment of ostracod valves with hydrogen peroxide before isotopic analyses was undertaken to remove soft part tissues or other organic matter and adhering sediment from the valves. The deviation of treated and untreated valves of the same individual was less than 0.2 ‰ for both δ^18^O and δ^13^C (Table 4; Fig. 8b) and remains in the same dimension as two untreated valves. Keatings et al. (2006) discussed the influence of common pre-treatment methods (including hydrogen peroxide) and their effect on stable isotope and trace-element analysis. Theses authors found deviations in the same range as in our study. In addition, there was no shift in the isotopic values observed when the period of treatment was extended. Hence, an influence of pre-treatment on the isotopic results can be excluded.

### Site specific ostracod isotope pattern

Various field studies demonstrated that the isotopic composition of ostracod valves is related to isotopic conditions of their host waters (Xia et al. 1997b; von Grafenstein et al. 1999; Keatings et al. 2002; Decrouy et al. 2011). In this study, isotopic values (δ^18^O and δ^13^C) of both *Cyprideis* species follow the trend of their host waters, despite the time lag between valve formation and sampling. *C. salebrosa* from Shell Creek in summer and winter are similarly low, while *C. salebrosa* from the Dominican Republic and *C. americana* from Jamaica furnish enriched δ^18^O and δ^13^C values (Fig. 9).

Wetterich et al. (2008) and Van der Meeren et al. (2011) studied the isotopic composition of ostracod valves from different lakes and ponds in comparison to simultaneous taken water samples. These authors also found a correlation of δ^18^O and δ^13^C values of ostracods to their host water in spite of the big time offset between valve calcification and sampling.

It is already known, that ostracods do not fractionate in equilibrium with their host water and most non-marine ostracods have a positive species specific offset (vital effect) to the water oxygen isotopes (Xia et al. 1997a; von Grafenstein et al. 1999; Keatings et al. 2002; Decrouy et al. 2011). Conditions during valve calcification are unknown for this study and a vital effect cannot be given. However, all four populations of *Cyprideis* exhibit a positive offset for oxygen isotopes ranging from +0.015 to +2.63 ‰ using the measured water data with an unknown time lag for a comparison to a calcite precipitated in their host water (Fig. 10a).

The comparison of δ^13^C values of ostracods and the DIC of their host water is even more complicated than for δ^18^O and also monthly measurements in lakes show just little correlations to isotopic measurements of ostracod valves (Decrouy et al. 2011). Several physical and biological processes including degassing, evaporation, photosynthesis and respiration influence the δ^13^C_DIC_ on different time scales (Leng and Marshall 2004). This is probably the reason for the positive and negative offset for the investigated samples (Fig. 10b) and the great range of values within the samples. However, carbon isotopes of ostracod valves in general are expected to be in equilibrium with an abiotic calcite (Keatings et al. 2002).

### Comparison of within sample variability of ostracod isotopes

The variability of single valve measurements of ostracod isotopes within a recent sample reveals information on changes in the environment during the period of valve formation. A high variation of the isotopic composition of the ostracods indicates, thus, stronger variation of their environment.

Although not knowing the exact life cycle of both species, we can assume that individuals with remaining soft part tissues were living at the time of sampling. Further, former studies on different *Cyprideis* species showed that their life cycle is finished within a year (e.g., Heip 1976; Schweitzer and Lohmann 1990; Marco-Barba 2010). Therefore, within sample variability reflect changes of the former year. Valves with no soft parts left may be older and reflect more than one generation. The high numbers of 42–79 measurements in our study (Table 5) are expected to be a representative dataset covering the last year.

The variation of δ^18^O is similar in Shell Creek and in Laguna del Rincon while the range of Parrotee Pond is twice as much (Fig. 9; Table 5). Considering just ‘fresh’ ostracods, the variation of δ^18^O values gets reduced for all sites except for FL_summer_ (Fig. 9b–f; Table 5).

Two factors influence the oxygen isotopes of ostracod valves: (1) the oxygen isotope composition of the water during calcification and (2) the temperature at that time. To calculate one of these two factors, the other one has to be known. Assuming a constant δ^18^O value, a temperature increase of 1 °C will lead to a decrease of 0.2 ‰ in the δ^18^O values of ostracod valves (Craig 1965; Bennett et al. 2011). The range of fresh ostracod δ^18^O values at the investigated sites then would reflect 9.8 °C in FL_summer_, 22.3 °C in FL_winter_, 14.6 °C in DR and to 27.6 °C in JM. The temperature range of FL_summer_ would lie within the annual temperature range of Schell Creek, but FL_winter_ has a much higher range that can only be explained by an additional change in the isotopic composition of the water. This could be achieved by a shift of the isotopic values of precipitation (Price et al. 2008) or the inflow of marine water during extreme high tide.

The annual air temperature range of the Dominican Republic and Jamaica only varies about 3.5 °C in both regions. The water temperature ranges in Laguna del Rincon and Parrotee Pond are probably higher, especially Parrotee Pond is shallow and water can heat and cool fast but the calculated temperature range from ostracod valves seems to big.

Medley et al. (2008) showed that Largo Enriquillo Valley is predominantly affected by changes in evaporation and precipitation during the Holocene. These authors reported δ^18^O values of *C. salebrosa* of the same range as in our study for the whole stratigraphic section. High isotopic values and the occurrence of irregular sieve pores were connected with an increase of salinity by high evaporation. Our data on noding indicates a higher salinity than observed during sampling. Together with the observed isotopic range this may indicate seasonal changes in the proportion of precipitation and evaporation.

Figure 9d shows two areas with a high density of isotopic values in the summer sample of Shell Creek of which one area overlaps with the majority of values from FL_winter_ (Fig. 9c).

These two areas in FL_summer_ may reflect two different generations of ostracods in the summer sample formed under different environmental conditions of which one represents the same generation of the former winter sample.

A temperature dependent isotopic difference would correspond with our size–temperature relationship and support the assumption of a life cycle with two maturations, one in summer (represented by FL_summer_) and one in late autumn (represented by FL_winter_) with the winter generation still living during the following summer. Nevertheless, if FL_summer_ represents two generations of *C. salebrosa* formed at different temperatures this difference is not reflected in a separation of sizes within the summer sample.

Schweitzer and Lohmann (1990) observed a developmental break for *Cyprideis* when water temperature drops below 15 °C. Water temperature of Shell Creek can reside around 15 °C in winter for several days (Kane and Dickman 2006). In this case, the development may be stopped or is at least reduced during winter.

This shift could also be caused by differences in the isotopic composition of the water. During the wet season the recharge of Shell Creek by precipitation is higher leading to lower δ^18^O values of the water. In this case, FL_summer_ may also contains two generations of *C. salebrosa* formed under different seasonal conditions.

Removing altered shells from the analyses, it is noticeable that particularly low values remain (Fig. 9). Most of the values of fresh *C. americana* valves from JM plot in the area of *C. salebrosa* from FL_winter_ (Fig. 9b). These valves may reflect just one generation of ostracods formed after the beginning of the wet season, when the isotopic values of Parrotee Pond can decrease.

The variation of δ^13^C in ostracod valves is dependent on changes in the δ^13^C_DIC_. The within sample variation of the δ^13^C values of *C. salebrosa* from Shell Creek is lower compared to the two other sites (Fig. 9). This can be explained by the permanent through-flow of water, which compensates high frequency fluctuations in the microenvironment in the littoral zone of the river (Atekwana and Krishnamurthy 1988). The variation of δ^13^C_DIC_ in the littoral zone of lakes can vary stronger than in rivers within a short time due to a missing mixture. Fast changes in photosynthesis and respiration can result in high isotopic variation (e.g., Leng and Marshall 2004).

## Conclusion

In this study we present data on valve size, node formation and isotopic signatures of valves from *Cyprideis salebrosa* and *Cyprideis americana* from Florida, Dominican Republic and Jamaica.

The distribution of the two *Cyprideis* species seems to be mainly controlled by the salinity of their host waters. *C. salebrosa* was found in a freshwater habitat (FL) and a habitat with slightly higher salinity values (DR) while *C. americana* (JM) occurred in water with a higher conductivity.

The valve size of *C. salebrosa* differs seasonally and regionally. The Dominican specimens were the smallest, correlated to a warmer climate. The specimens from Florida show a seasonal size difference with smaller individuals in summer when temperatures are higher. The investigated salinity range is too small to reveal information on its influence on the valve size of *C. salebrosa.*

The node position of *C. salebrosa* has been reported earlier while this is the first report of the node position of *C. americana*. Less noding of *C. salebrosa* form the Dominican Republic indicates higher salinity during valve formation compared to Floridian specimens.

Differences in the isotopic composition of the valves of *C. salebrosa* and *C. americana* refer to site specific water conditions.

The δ^18^O values of *Cyprideis* correspond in general with the isotopic composition of their host water with a positive offset at all sites. Shell Creek, Florida, reflects rainfall influenced isotopic oxygen signatures resulting in low values while values of Laguna del Rincon, Dominican Republic, and Parrotee Pond, Jamaica, are enriched in δ^18^O due to longer residence time. Higher values of δ^13^C can be explained by equilibration with atmospheric CO_2_ in Laguna del Rincon and Parrotee Pond.

Within sample variability of ostracod stable isotopes are considered to reflect hydrological and/or seasonal changes during the past year. Changes in the water conditions during valve formation lead to different variations of the isotopic values of *Cyprideis* at the sites. Temperature differences only cannot explain the variation of the site. For Shell Creek a change in the isotopic composition of precipitation is considered (change in vapor source) while in Laguna del Rincon and Parrotee Pond a change in the proportion of precipitation and evaporation may explain the high variability. Less noding of *C. salebrosa* for Laguna del Rincon may be also connected to higher salinity at the time of valve calcification due to higher evaporation during that time.

A separation of the δ^18^O values of living *C. salebrosa* from the summer sample of Shell Creek into two groups indicates a life cycle with two maturations during 1 year. Valve size analyses do not reflect this pattern.

The variation of the δ^13^C values of *Cyprideis* is controlled by fast periodic changes in their microhabitat. The permanent stream flow of Shell Creek prevents the accumulation of heavy ^13^C in the littoral zone of the river resulting in a low variation in δ^13^C values of *C. salebrosa*. The restricted mixture in Laguna del Rincon and Parrotee Pond leads to high temporary fluctuations of the δ^13^C values of ostracod valves by changes in the biological activity.

Although the number of investigated sites is small, the high number of investigated individuals within the samples reveals information on the plasticity of morphological and isotopic composition of the *Cyprideis* species.
